# Notable Increasing Trend in Azole Non-susceptible *Candida tropicalis* Causing Invasive Candidiasis in China (August 2009 to July 2014): Molecular Epidemiology and Clinical Azole Consumption

**DOI:** 10.3389/fmicb.2017.00464

**Published:** 2017-03-22

**Authors:** Xin Fan, Meng Xiao, Kang Liao, Timothy Kudinha, He Wang, Li Zhang, Xin Hou, Fanrong Kong, Ying-Chun Xu

**Affiliations:** ^1^Department of Clinical Laboratory, Peking Union Medical College HospitalBeijing, China; ^2^Graduate School, Peking Union Medical College, Chinese Academy of Medical SciencesBeijing, China; ^3^Department of Clinical Laboratory, First Affiliated Hospital of Sun Yat-Sen UniversityGuangzhou, China; ^4^Charles Sturt University, Leeds ParadeOrange, NSW, Australia; ^5^Centre for Infectious Diseases and Microbiology Laboratory Services, ICPMR–Pathology West, Westmead Hospital, University of SydneySydney, NSW, Australia

**Keywords:** *Candida tropicalis*, invasive candidiasis, antifungal susceptibility, azole resistance, genotyping, China

## Abstract

**Objectives:** To report the notable increasing trends of *C. tropicalis* antifungal resistance in the past 5 years, and explore molecular epidemiology, and the relationship between clinical azoles consumption and increased resistance rate.

**Methods:** Between August 2009 and July 2014, 507 non-duplicated *C. tropicalis* isolates causing invasive candidiasis were collected from 10 hospitals in China. The *in vitro* antifungal susceptibility of nine common agents was determined by Sensititre YeastOne™ using current available species-specific clinical breakpoint (CBPs) or epidemiological cut-off values (ECVs). A high discriminatory three-locus (ctm1, ctm3, and ctm24) microsatellite scheme was used for typing of all isolates collected. Clinical consumption of fluconazole and voriconazole was obtained and the Defined Daily Dose measurement units were assigned to the data.

**Results:** Overall, 23.1 and 20.7% of isolates were non-susceptible to fluconazole and voriconazole, respectively. And over 5 years, the non-susceptible rate of *C. tropicalis* isolates to fluconazole and voriconazole continuously increased from 11.2 to 42.7% for fluconazole (*P* < 0.001), and from 10.4 to 39.1% for voriconazole (*P* < 0.001). Four genotype clusters were observed to be associated with fluconazole non-susceptible phenotype. However, the increase in azole non-susceptible rate didn't correlate with clinical azole consumption.

**Conclusions:** The rapid emergence of azole resistant *C. tropicalis* strains in China is worrying, and continuous surveillance is warranted and if the trend persists, empirical therapeutic strategies for *C. tropicalis* invasive infections should be modified.

## Introduction

*C. tropicalis* is an important pathogen causing invasive candidiasis (IC), particularly in patients with cancer and leukemia (Munoz et al., 2011). Worldwide, *C. tropicalis* has become the first to fourth leading cause of IC in different geographic regions (Munoz et al., 2011; Wang et al., 2012; Pfaller et al., 2015). Furthermore, resistance to azoles, particularly to fluconazole, is increasingly being reported in *C. tropicalis* isolates (Kothavade et al., 2010).

The CHIF-NET study, a surveillance program for invasive yeast infections including IC in China, has provided much informative data on nationwide epidemiology and antifungal susceptibility of pathogens since its inception in August 2009 (Wang et al., 2012; Xiao et al., 2015). A close look at the results for the first 3 years (to July 2012) of the surveillance program showed a small but gradual decrease in the rate of *C. tropicalis* susceptibility to azole drugs, which was not significant (Xiao et al., 2015). However, in the fourth and fifth years (2013 and 2014), the situation had significantly worsened, with the rate of azole non-susceptibility increasing rapidly. To bring awareness to our domestic and international colleagues, we hereby report our detailed findings on the trends of *C. tropicalis* antifungal susceptibility from the CHIF-NET study in the past 5 years, and explore molecular epidemiology and any relationship between clinical azole consumption and increased resistance rate.

## Materials and methods

### Isolates

**Figure 1:**
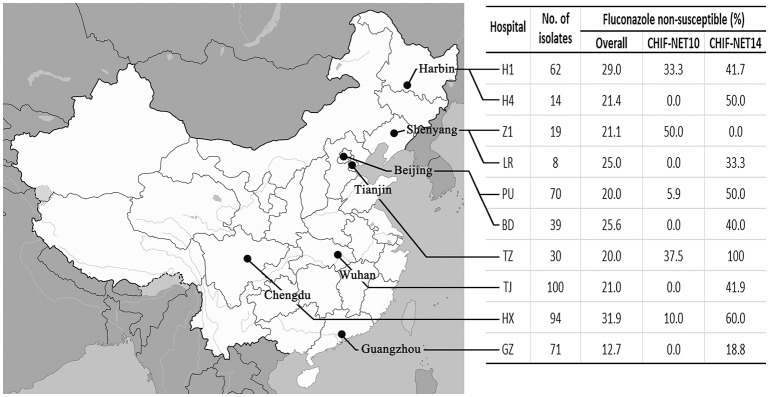
**Geographic distribution of the 10 surveillance centers involved in this study, number of isolates collected, and change of fluconazole non-susceptible rate from the first to the last surveillance year in each center**.

Non-duplicated *C. tropicalis* isolates included in this study were collected consecutively from unique patients under the CHIF-NET program during a 5-year period from August 1st, 2009 to July 31st, 2014. The program was approved by the Human Research Ethics Committee of Peking Union Medical College Hospital (S-263). The study inclusion criteria was described previously (Wang et al., 2012) and all isolates that met the criteria were forwarded to a central laboratory (the Department of Clinical Laboratory, Peking Union Medical College Hospital) for molecular identification confirmation and antifungal susceptibility testing following a standardized study protocol (Wang et al., 2012). To ensure coherence and consistency of surveillance data over time, only *C. tropicalis* isolates from 10 hospitals that consistently participated in the study over 5 years, were included in the present study (Figure 1, see Acknowledgements for the participated hospitals).

### Antifungal susceptibility testing

The *in vitro* susceptibility of isolates to nine antifungal drugs—fluconazole, voriconazole, itraconazole, posaconazole, caspofungin, micafungin, anidulafungin, amphotericin B, and 5-flucytosine—was determined using Sensititre YeastOne™ YO10 methodology (Thermo Scientific, Cleveland, Ohio, USA), following the manufacturer's instructions. For each run, the quality control strains were *Candida parapsilosis* ATCC 22019 and *Candida krusei* ATCC 6258. Current available species-specific clinical breakpoint (CBPs) or epidemiological cut-off values (ECVs) were used for interpretation of results (Table 1; Canton et al., 2012; CLSI, 2012).

**Table 1:** **Clinical breakpoints (CBPs), epidemiologic cut-off values (ECVs), and susceptibility results among 585 *Candida tropicalis* isolates studied**.

**Antifungal agents**	**MIC (mg/L)**			**Category (%)**			**CPBs (mg/L)**				**ECVs (mg/L)**
	**50%**	**90%**	**GM**	**S/WT**	**SDD/I**	**R/NWT**	**S**	**SDD**	**I**	**R**	
Fluconazole	2	32	2.59	76.9	10.3	12.8	≤2	4	–	≥8	–
Voriconazole	0.12	1	0.13	79.3	9.3	11.4	0.125	0.25–0.5	–	≥1	–
Itraconazole	0.25	0.5	0.21	100.0	–	0.0	–	–	–	–	1
Posaconazole	0.12	0.5	0.17	100.0	–	0.0	–	–	–	–	2
Caspofungin	0.03	0.06	0.04	99.6	0.0	0.4	≤0.25	–	0.5	≥1	–
Micafungin	0.03	0.03	0.03	99.6	0.0	0.4	≤0.25	–	0.5	≥1	–
Anidulafungin	0.06	0.25	0.07	99.2	0.4	0.4	≤0.25	–	0.5	≥1	–
5-Flucytosine	0.03	0.12	0.07	99.4	–	0.6	–	–	–	–	0.5
Amphotericin B	1	1	0.75	100.0	–	0.0	–	–	–	–	2

*GM, geometric mean; S, susceptible; SDD, susceptible dose-dependent; I, intermediate; R, resistant; WT, wild-type; NWT, non-wild-type*.

### Three-locus microsatellite genotyping

A high discriminatory three-locus (ctm1, ctm3, and ctm24) microsatellite scheme was used for typing of all isolates collected as previously described (Fan et al., 2016). A minimum spanning tree (MST) was drawn by BioNumerics software v7.5 (Applied Maths, Austin, TX) to illustrate phylogenetic relatedness among isolates and correlation between microsatellite genotypes and azole-resistance phenotypes.

### Clinical antifungal consumption

Clinical consumption of fluconazole and voriconazole was obtained and the Defined Daily Dose (DDD) measurement units [Anatomical Therapeutic Chemical (ATC) /DDD version 2007] were assigned to the data. The DDD per 100 patient-days in hospitals was used to measure time trends.

### Data analysis

All statistical analyses were performed using IBM SPSS software (version 22.0; IBM SPSS Inc., New York, USA). Categorical variables were compared using the Chi-square or Fisher's exact test, and continuous variables by the Mann–Whitney *U*-test. The relationship between antifungal usage and the incidence of antifungal susceptibility was determined using Spearman's coefficient for non-parametric correlation. A *P* < 0.05 was considered significant.

## Results

### *In vitro* susceptibility to azoles

A total of 507 *C. tropicalis* isolates were collected from 10 surveillance centers over 5 years (ranging from 89 to 115 isolates every year), which accounted for 15.7% of all *Candida* isolates collected. Overall, 23.1 and 20.7% of isolates were non-susceptible to fluconazole and voriconazole, respectively (Table 1), with 11.4% of the isolates showing cross-resistance to both.

**Figure 2:**
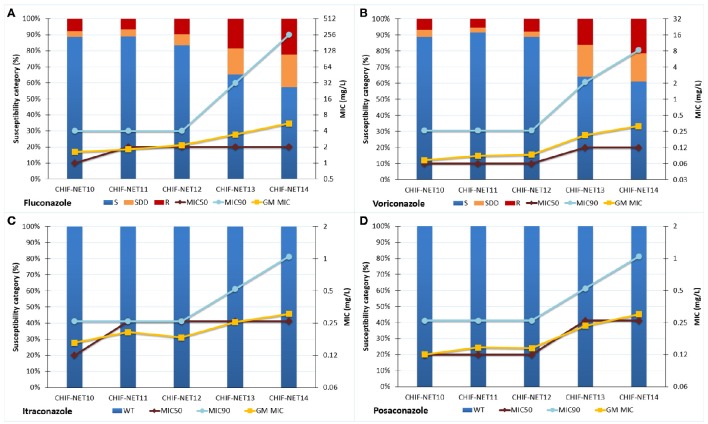
**Trends of susceptibility (including susceptible or wild-type rate, and MIC_50_, MIC_90_, and GM MIC values) of 507 *C. tropicalis* isolates to four azoles (A)**, fluconazole; **(B)**, voriconazole; **(C)**, itraconazole; **(D)**, posaconazole, over 5 years. S, susceptible; SDD, susceptible dose-dependent; R, resistant; WT, wild-type; NWT, non-wild-type; MIC, minimum inhibitory concentration; GM, geometric mean.

Over 5 years, there was a significant increase in azole non-susceptibilities, particularly during the last 2 years. As shown in Figure 2, the non-susceptible rate of *C. tropicalis* isolates to fluconazole and voriconazole continuously increased from 11.2 to 42.7% for fluconazole (*P* < 0.001), and from 10.4 to 39.1% for voriconazole (*P* < 0.001), with the rate accelerating in the fourth (2013) and fifth (2014) surveillance years. The prevalence of fluconazole-voriconazole cross-resistant isolates also increased from 6.6 to 21.7% (*P* < 0.001). In addition, the fluconazole and voriconazole MIC_50_ and geometric mean (GM) MIC values in the fifth year were 1–3-fold higher than those in the first year, while the MIC_90_ values also notably increased by over 5 fold. Moreover, although all isolates remained of wild-type phenotype to itraconazole and posaconazole, the GM MIC and MIC_50_ values for these two drugs also rose by over 2 fold, and the MIC_90_ values had a 4-fold increase during the study period (Figure 2).

Of note, the increase in azole non-susceptibility occurred in all the participating hospitals (fluconazole non-susceptible rate increased by 8.4–62.5%) except for one (Z1) hospital (the rate decreased by 50.0%; Figure 1), and was significant amongst all wards (non-susceptible rate increased by 22.3–71.4%), all specimen types (non-susceptible rate increased by 11.1–60.0%; Table 2). The fluconazole non-susceptible rate was lowest in patients <18 year-old (19.2%), and the non-susceptible rates increased with increasing age (Table 2), although statistically insignificant (*P* >0.05). However, the fluconazole non-susceptible rates were increased significantly amongst all patient age groups over the 5 years (rate increased from 25.0 to 33.8%; Table 2).

**Table 2:** **Trends of fluconazole non-susceptible (Flu NS) rate among *C. tropicalis* isolates by wards and specimen types during 5 years**.

**Characters**	**Overall**		**CHIF-NET10**		**CHIF-NET11**		**CHIF-NET12**		**CHIF-NET13**		**CHIF-NET14**		**P value^a^**
	***n***	**Flu NS%**	***n***	**Flu NS%**	***n***	**Flu NS%**	***n***	**Flu NS%**	***n***	**Flu NS%**	***n***	**Flu NS%**	
Overall	507	23.1	89	11.2	108	11.1	115	16.5	92	34.8	103	42.7	<0.001
**WARDS**													
Outpatient/Emergency	36	22.2	7	14.3	8	25.0	9	0.0	7	42.9	5	40.0	ND^b^
Inpatient	471	23.1	82	11.0	100	10.0	106	17.9	85	34.1	98	42.9	<0.001
Surgery	125	20.0	25	4.0	22	4.5	30	16.7	21	38.1	27	37.0	0.004
Internal medicine	147	21.8	23	8.7	35	11.4	27	18.5	33	36.4	29	31.0	0.049
Intensive care unit	169	25.4	31	19.4	36	11.1	40	15.0	27	33.3	35	51.4	0.007
Other wards^c^	30	30.0	3	0.0	7	14.3	9	33.3	4	0.0	7	71.4	ND^b^
**SPECIMEN TYPES**													
Blood	220	27.3	36	19.4	44	9.1	45	11.1	49	44.9	46	47.8	0.008
Ascitic fluid	130	18.5	24	4.2	22	4.5	35	22.9	18	16.7	31	35.5	0.005
Bronchoalveolar lavage fluid	36	33.3	4	0.0	13	23.1	10	40.0	4	50.0	5	60.0	ND^b^
Pus	29	24.1	6	33.3	6	0.0	3	0.0	5	20.0	9	44.4	ND^b^
Bile	27	14.8	6	0.0	5	0.0	10	20.0	1	0.0	5	40.0	ND^b^
Other specimens^d^	65	27.3	13	0.0	18	22.2	12	0.0	15	26.7	7	28.6	ND^b^
**AGE (YEAR)**													
0–18	26	19.2	3	0.0	4	25.0	6	0.0	9	33.3	4	25.0	ND^b^
19–45	138	19.6	26	11.5	26	11.5	35	8.6	21	28.6	30	40.0	0.016
46–64	183	23.5	30	10.0	44	11.4	38	21.1	36	33.3	35	42.9	0.003
65 and above	160	26.3	30	13.3	34	8.8	36	22.2	26	42.3	34	47.1	0.004

a Statistical analysis for fluconazole non-susceptible rate of CHIF-NET14 vs. CHIF-NET10

b *ND, not done because of small sample size*.

c *Including gynecology, pediatric, geriatric and dermatology wards*.

d *Including pleural fluid, venous catheter, cerebrospinal fluid, tissue and peritoneal dialysate fluid*.

### Microsatellite genotyping and phylogenetic analysis

**Figure 3:**
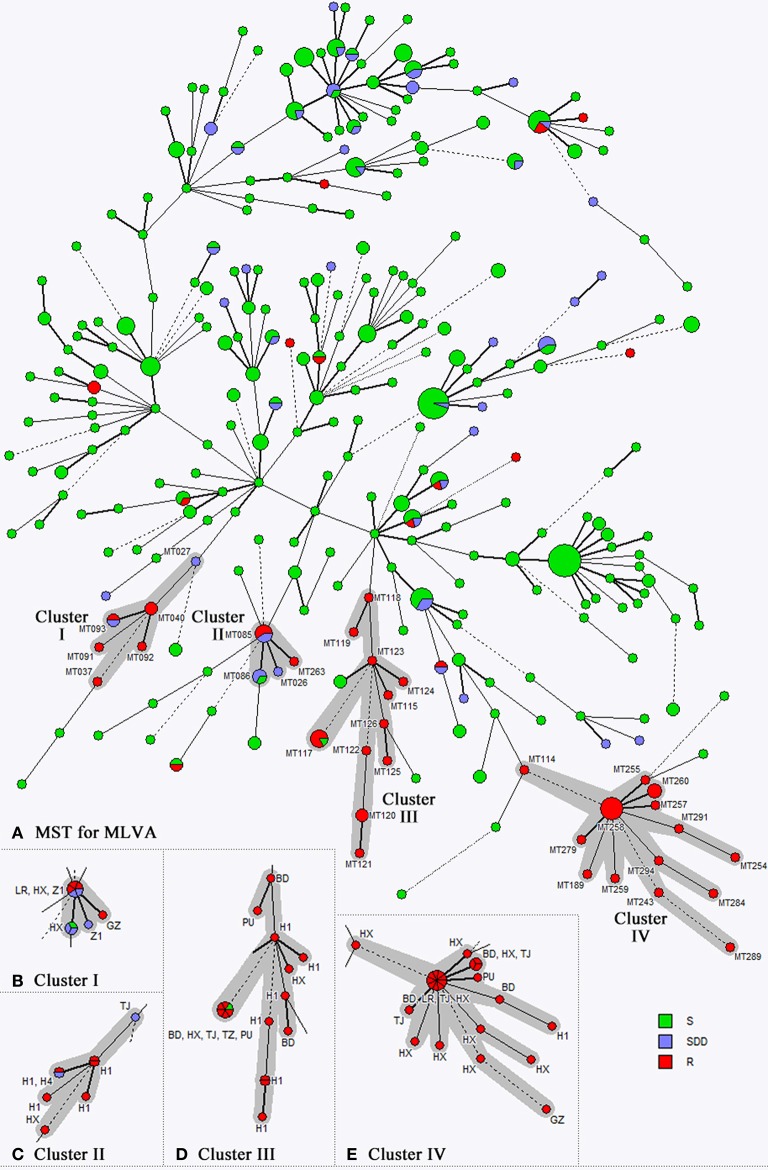
**The minimum spanning tree (MST) draw by three-locus microsatellite genotyping results of 507 *C. tropicalis* isolates**. Panel **(A)** and four genotype clusters associated with fluconazole non-susceptible phenotypes **(B–E)**. Each circle corresponds to a microsatellite genotype, and the size of circle represents number of isolates for each genotype. Different colors in the circle represents different fluconazole susceptibility categories. The lines between circles indicate the similarity between profiles.

By using the three-locus microsatellite scheme, 296 genotypes were identified amongst the 507 isolates studied (Figure 3A). The most common genotype was MT178 (21/507 isolates, 4.1%), followed by MT043 (19/507 isolates, 3.7%), and no other genotypes comprised >10 isolates (<2% of all isolates studied). The MST analysis showed that 57 of 117 (48.7%) fluconazole non-susceptible isolates were embedded in four genotype clusters (Figures 3A–E). The biggest cluster associated with fluconazole non-susceptible phenotype was cluster IV, which comprised 24 isolates (20.5% of 117 fluconazole non-susceptible isolates, and 4.7% of all isolates studied) of 14 genotypes (Figure 3E). Moreover, all cluster IV *C. tropicalis* isolates were fluconazole and voriconazole cross-resistant, and no fluconazole susceptible isolates were observed within this cluster. In addition, 16 fluconazole resistant isolates (13.7% of 117 fluconazole non-susceptible isolates, and 3.2% of all isolates studied) from 11 genotypes belonged to cluster III (Figure 3D). Clusters I and II were comparably small, and compromised of eight and nine fluconazole non-susceptible isolates, respectively (Figures 3B,C). Other fluconazole non-susceptible isolates were scattered in the MST (Figure 3A). Of note, there was no correlation observed between geographic regions and fluconazole non-susceptible clustered cases (Figures 3B–E).

### *In vitro* susceptibility to echinocandins, amphotericin B, and 5-flucytosine

For non-azole antifungal agents, all *C. tropicalis* isolates in the present study were of wild-type to amphotericin B, and only 0.7% of isolates were of non-wild type phenotype to 5-flucytosine. Over 99% of the isolates remained susceptible to all three echinocandins tested. In addition, during the 5 years of surveillance, there were no significant changes (within ± 1 dilution) in MIC_50_, MIC_90_, and GM MIC values for these drugs.

### Clinical azole consumption and correlation with azole susceptibility

**Figure 4:**
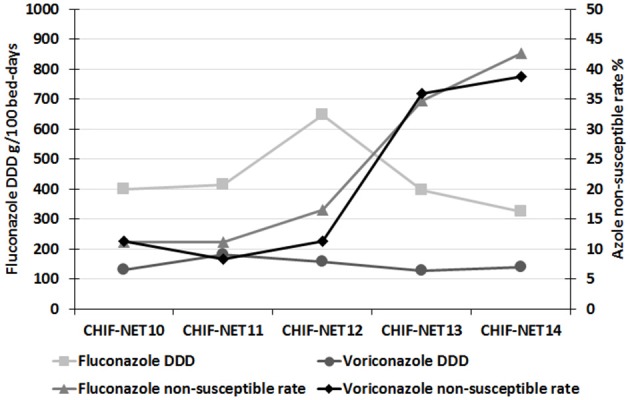
**Trends of fluconazole and voriconazole susceptibilities and clinical consumption of these two drugs over the 5-year surveillance period**.

At nine hospitals, the usage of fluconazole and voriconazole (DDD) were 393 and 151 g per 100 patient-days, respectively over 5 years. The use of voriconazole was generally stable over 5 years (varied from 126 to 181 g per 100 patient-days; Figure 4). However, the use of fluconazole increased from 398 g per 100 patient-days from the first year to 647 g per 100 patient-days in the third year, but decreased since then to 323 g per 100 patient-days in the fifth year (Figure 4). There was no significant correlation between the use of fluconazole or voriconazole and prevalence of azole non-susceptible isolates analyzed by year (*P* > 0.05).

## Discussion

*C. tropicalis* has become a predominant non-*albicans Candida* species causing IC worldwide, and its prevalence varies across geographic regions (Wang et al., 2012; Pfaller et al., 2015; Xiao et al., 2015). For example, in European countries, *C. tropicalis* is the second to third commonest non-*albicans Candida* species (Pfaller et al., 2010, 2015), whilst in India the species causes more candidemia cases (about 40%) than *C. albicans* (Chander et al., 2013). According to data obtained in this study and from previous studies, *C. tropicalis* is the third commonest pathogenic *Candida* species in China, and its prevalence has generally been stable (15–20%; Wang et al., 2012; Liu et al., 2014; Xiao et al., 2015). In addition, some previous studies indicated that invasive infections caused by *C. tropicalis* have higher mortality compared to those caused by other non-*tropicalis Candida* species (Montagna et al., 2013; Andes et al., 2016).

Worldwide, among commonest *Candida* species causing IC, *C. albicans* and *C. parapsilosis* remained susceptible to azoles (fluconazole resistant rate <3%); however, it has been widely noticed that *C. glabrata* had notably high resistant rate to azole agents (fluconazole resistant rate >12%; Xiao et al., 2015; Castanheira et al., 2016). Although there is a general consensus worldwide that *C. tropicalis* strains exhibit a moderate level of azole resistance (Kothavade et al., 2010; Jiang et al., 2013; Guinea et al., 2014), it is important to note that the rates of *C. tropicalis* resistance to azoles in North America and most European countries are low. For example, *C. tropicalis* resistant rates to fluconazole in the United States are generally <7% (Lockhart et al., 2012; Pfaller et al., 2015), whilst those reported in European countries vary from 0 to 12% (Orasch et al., 2014; Minea et al., 2015; Posteraro et al., 2015; Tadec et al., 2016).

However, our present study highlights a sharp increase in the prevalence of fluconazole and voriconazole non-susceptible *C. tropicalis* isolates in China, particularly since 2013. In addition, although all the isolates were of wild type phenotype to posaconazole and itraconazole interpreted by previously proposed ECVs, the MIC_50_s, MIC_90_s, and GM MICs of all four azole drugs tested continuously increased in the fourth and fifth years. Specifically, four azoles MIC_90_s in the last year was 4–6-fold higher than in the first year. Moreover, the notable increase in the rate of fluconazole and voriconazole non-susceptibility was observed in nine of ten hospitals, and amongst all wards, specimen types and patient age groups, which indicates a widespread phenomenon in China.

The high fluconazole non-susceptible rate amongst *C. tropicalis* has also been noted in a few previous clinical surveillance studies in Asia-Pacific regions. For instance, the global SENTRY surveillance reported an overall fluconazole non-susceptible rate of 11.6% amongst *C. tropicalis* isolates collected from 31 countries in 2013. The majority (81.8%) of these non-susceptible isolates were from Asia-Pacific regions, and 31.8% were from China (Castanheira et al., 2016). Another multicenter study involving13 centers from Asia-Pacific regions in 2012–2014, also reported a high *C. tropicalis* non-susceptible rate of 24.2% (Tan et al., 2016). No data from China was included in that study.

Further, we performed molecular typing and phylogenetic analysis for all *C. tropicalis* isolates collected. A highly discriminatory microsatellite typing assay was employed (Fan et al., 2016), and four genotype clusters associated with fluconazole non-susceptible phenotypes were revealed. The four clusters comprised 48.7% of the fluconazole non-susceptible isolates overall. Previous studies in Taiwan also observed regional dissemination of genetically close-related fluconazole-resistant *C. tropicalis* isolates (Chou et al., 2007; Li et al., 2009). Therefore, continuous monitoring of the fluconazole non-susceptible *C. tropicalis* isolates assisted by molecular typing assays is warranted.

Although the usage of azoles, especially fluconazole, has been considered an important factor contributing to the increasing prevalence of azole less-susceptible *Candida* species (Miceli et al., 2011; Won et al., 2015; Jensen, 2016), findings from the present study seem not to support this in China. Specifically, fluconazole consumption density was higher in the first 3 years but then decreased in the last 2 years. This was most likely a result of a sustained official campaign by the Chinese government on antimicrobial and antifungal stewardship initiated in 2011 (Xiao et al., 2013). However, the increasing trend in azole non-susceptible *C. tropicalis* in China still calls for attention in future clinical use of azoles. Infectious Diseases Society of America has recommended echinocandin as first-line drugs against *C. glabrata* candidiasis, as the species has high azole resistant rates (Pappas et al., 2009). As the *C. tropicalis* isolates collected in the present study, including those azole non-susceptible strains, remained highly susceptible to echinocandin drugs, the echinocandins could be effective clinical alternatives in China. However, acquired resistance has been observed after echinocandins exposures (Matsumoto et al., 2014; Delliere et al., 2016; Bordallo-Cardona et al., 2017). Therefore, it's also necessary to maintain continuous monitoring of echinocandin susceptibility of *C. tropicalis* in China.

In addition to human clinical practice, the role of contaminated environment and veterinary usage of antimicrobials and antifungals also raised great global concerns on accelerating the emergence and spread of drug-resistance. A recent study from Brazil in 2015 has reported a 47% fluconazole resistant rate in *C. tropicalis* from veterinary sources (Cordeiro Rde et al., 2015). In addition, a research from Taiwan observed genetically related fluconazole non-susceptible *C. tropicalis* isolates from human hosts and environmental soil samples (Yang et al., 2012). Therefore, although no environmental or veterinary *C. tropicalis* isolates were obtained in China for antifungal susceptibility assessments and phylogenetic relatedness analysis against the clinical isolates in this study, the impact of non-clinical human practice and environment dissemination routes toward *C. tropicalis* isolates, especially those azole non-susceptible ones, cannot be overlooked.

## Conclusion

Our findings show an unusual high-level of fluconazole and voriconazole resistance, and a significant trend of increasing azole non-susceptibility among *C. tropicalis* isolates from IC in China for the period August 2009 to July 2014, which is particularly notable during the last 2 years. Several genotype clusters were observed to be associated with fluconazole non-susceptible phenotype, but the increase in fluconazole non-susceptible rate didn't correlate with clinical azole use in these hospitals. Continuous surveillance and molecular epidemiology study is still warranted, and if the trend persists, empirical therapeutic strategies for *C. tropicalis* invasive infections should be modified.

## Author contributions

XF, MX, KL, and YC conceived the work. XF, KL, HW, LZ, and XH performed the experiments. XF and MX performed the data analysis. XF, MX, TK, and FK drafting the manuscript. All authors participated in the critical review of this manuscript.

## Funding

This study was supported by grants from the Research Special Fund for Public Welfare Industry of Health (grant no. 201402001) and CAMS Innovation Fund for Medical Sciences (grant no. 2016-I2M-1-014).

### Conflict of interest statement

The authors declare that the research was conducted in the absence of any commercial or financial relationships that could be construed as a potential conflict of interest.
